# Assessing self‐reported prolonged grief disorder with “clinical checks”: A proof of principle study

**DOI:** 10.1002/jts.23100

**Published:** 2024-08-19

**Authors:** Mark Shevlin, Philip Hyland, Marylène Cloitre, Chris Brewin, Dmytro Martsenkovskyi, Menachem Ben‐Ezra, Kristina Bondjers, Thanos Karatzias, Michael Duffy, Enya Redican

**Affiliations:** ^1^ School of Psychology Ulster University Coleraine Northern Ireland; ^2^ Department of Psychology Maynooth University Kildare Ireland; ^3^ National Center for PTSD Dissemination and Training Division VA Palo Alto Health Care System Palo Alto California USA; ^4^ Department of Psychiatry and Behavioral Sciences Stanford University Stanford California USA; ^5^ Department of Clinical, Educational and Health Psychology University College London London UK; ^6^ Department of Psychiatry and Narcology Bogomolets National Medical University Kyiv Ukraine; ^7^ SI Institute of Psychiatry Forensic Psychiatric Examination and Drug Monitoring of the Ministry of Health of Ukraine Kyiv Ukraine; ^8^ School of Social Work Ariel University Ariel Israel; ^9^ Norwegian Centre for Violence and Traumatic Stress Studies Oslo Norway; ^10^ School of Health & Social Care Edinburgh Napier University, Edinburgh Scotland UK; ^11^ School of Social Sciences, Education and Social Work Queens University Belfast Belfast Northern Ireland

## Abstract

Psychological assessment is commonly conducted using either self‐report measures or clinical interviews; the former are quick and easy to administer, and the latter are more time‐consuming and require training. Self‐report measures have been criticized for producing higher estimates of symptom and disorder presence relative to clinical interviews, with the assumption being that self‐report measures are prone to Type 1 error. Here, we introduce the use of “clinical checks” within an existing self‐report measure. These are brief supplementary questions intended to clarify and confirm initial responses, similar to what occurs in a clinical interview. Clinical checks were developed for the items of the International Grief Questionnaire (IGQ), a self‐report measure of *ICD‐11* prolonged grief disorder (PGD). Data were collected as part of a community survey of mental health in Ukraine. Individual symptom endorsements for the IGQ significantly decreased with the use of clinical checks, and the percentage of the sample that met the *ICD‐11* diagnostic requirements for PGD fell from 13.6% to 10.2%, representing a 24.8% reduction in cases. The value and potential broader application of clinical checks are discussed.

Self‐report questionnaires provide a quick and inexpensive way to assess psychopathology. For example, the presence and severity of generalized anxiety disorder (GAD), major depressive disorder (MDD), and posttraumatic stress disorder (PTSD) can be assessed in a few minutes using measures like the Generalized Anxiety Disorder Questionnaire–7 (GAD‐7; Spitzer et al., 2006), Patient Health Questionnaire–9 (PHQ‐9; Kroenke et al., 2001), and the International Trauma Questionnaire (ITQ; Cloitre et al., 2018), respectively. However, self‐report instruments typically generate severity and prevalence figures that are higher than those produced when using clinical interviews, and the assumption is that self‐report measures are prone to Type 1 (i.e., false‐positive) errors (e.g., Gelezelyte et al., 2022; Kertz, et al., 2013; Levis et al., 2020; Linscott & van Os, 2013; Stevens et al., 2013). A recent example to illustrate the point comes from Gelezelyte et al. (2022), who compared self‐report and structured interview measures of complex PTSD (CPTSD), as defined in the *International Statistical Classification of Diseases and Related Health Problems* (11th ed.; *ICD‐11*; World Health Organization, 2019), and reported lower endorsement rates for all symptom clusters and the disorder based on the clinical interview. We calculated the relative percentage decreases for symptom endorsement and disorder prevalence based on Gelezelyte et al.’s (2022) findings; symptom decreases ranged from 16.6% (avoidance) to 61.6% (emotional numbing), with a relative decrease of 44.8% for CPTSD.

It is often, but not always, the role of the clinical interviewer to provide a “quality control” check on an interviewee's response. The interviewer can follow up on initial responses to gather additional information, clarify potential misinterpretations, provide explanations about what constitutes “symptomatic” levels of distress, and/or ensure that specific criteria (e.g., time, relation to a specific event) are being adhered to. A related function of the interviewer is to try to ensure that normative experiences, or those that do not meet severity or frequency requirements, are not considered to be clinically significant problems. In this way, they conduct and provide “clinical checks”

The absence of any such checks in a self‐report measure may be the greatest weakness of this assessment method. This study is, therefore, a proof of principle investigation that aimed to test whether clinical checks can be embedded into a self‐report measure to reduce reported symptom endorsements and overall diagnostic prevalence estimates, which may be indicative of a reduction in false positives. If so, this would represent the first step in integrating these checks—a strength of clinical interviews—into self‐report questionnaires. In this context, we use the term “clinical checks” to describe simple follow‐up questions intended to ensure that the respondent accurately understood the meaning of the symptom indicator question and confirm the initial response. The rationale is that it would be desirable to get an important benefit of a clinical interview for a small increase in the time and demand associated with a self‐report assessment. We focused on developing a set of clinical checks for the items of the International Grief Questionnaire (IGQ: Hyland et al., 2024), a recently developed measure of *ICD‐11* prolonged grief disorder (PGD). This was considered an appropriate target measure as bereavement is often associated with emotional distress, and it is critical—and challenging—to differentiate “normal” grief from “pathological” or clinically relevant grief (Eisma, 2023). We hypothesized that there would be statistically significant reductions in both symptom endorsements and the proportion of people who met the diagnostic requirements for *ICD‐11* PGD when clinical checks were used compared to when they were not used.

## METHOD

### Participants and procedure

This study is based on data collected from September 7, 2023, to September 18, 2003, as part of the Mental Health of Parents and Children in Ukraine Study: 2023 Follow‐Up. Participants were recruited from an existing panel of research participants that was, prewar, nationally representative based on the most recent Ukrainian census data. We used nonprobability quota sampling methods to construct a sample that was as representative of the adult population of Ukraine as possible given the current circumstances. The inclusion criteria were being 18 years of age or older at the time of the survey, living in Ukraine, and being capable of completing the survey in Ukrainian. Quota variables used to construct the sample were sex, age, and region of Ukraine. Full details of the survey and mental health of participants are available in Martsenkovskyi et al. (2024). Ethical approval for the project was obtained from the SI Institute of Psychiatry, Forensic Psychiatric Examination, and Drug Monitoring of the Ministry of Health of Ukraine.

### Measures

The IGQ (Hyland et al., 2024) and IGQ–Clinical Checks (IGQ‐CC) are self‐report measures that can be used to capture all diagnostic requirements for *ICD‐11* PGD. Participants were initially screened for lifetime bereavement (“During your life has someone close to you died [e.g., a partner, parent, child, close friend?]”), their relationship to the deceased, and how long ago the bereavement occurred. If a participant reported multiple bereavements, they were asked to pick the bereavement that has caused them the most distress when answering all additional questions.

The IGQ includes five items: Two items measuring the two “core” *ICD‐11* PGD symptoms (i.e., yearning or longing for the deceased and a preoccupation with the deceased) and three items assessing associated symptoms that capture emotional distress related to the loss (all items can be found in Table 1). Participants were instructed to indicate how bothered they had been by each symptom in the last week, using a 5‐point Likert scale with response options of 0 (*not at all*), 1 (*a little bit*), 2 (*moderately*), 3 (*quite a bit*), and 4 (*extremely*). By convention across all *ICD‐11* stress‐related disorders, a symptom is considered “present” based on a score of 2 or higher on the Likert scale. In the present sample, internal reliability for IGQ scores was good, Cronbach's α = .86.

**TABLE 1:** International Grief Questionnaire (IGQ) items, clinical checks, and rationale

Clinical check	Rationale
*IGQ Item 1: Yearning/longing for the deceased almost every day?*	
This is more than just missing your loved one. It is an intense and painful desire to be with the deceased again. Is this what you felt almost every day over the past week?	Stresses that missing the deceased is insufficient, and this has to be an intense and painful experience. The “almost every day over the past week” qualifier emphasizes the *ICD‐11* requirement for a “persistent and pervasive grief response.”

*IGQ Item 2: Thinking too much about the deceased almost every day?*	
This means thinking so much about your lost loved one that it causes you pain and interferes with you doing other things. Is this what you experienced almost every day in the past week?	Clarifies the impairing and negative nature of this experience. It also stresses the *ICD‐11* requirement for “*persistent* preoccupation,” which is a feature of the two core PGD symptoms.

*IGQ Item 3: Feeling guilty or angry about my loss*.	
Do you feel like this frequently and does it cause you distress?	Clarifies that guilt and anger should be a regular experience that is distressing in nature rather than something fleeting and/or nondistressing.

*IGQ Item 4: Having trouble accepting the death of my loved one*.	
This means that you sometimes find it difficult to come to terms with the fact that your loved one has died, and you wish it were not the case. Is this what you have been experiencing?	Intended to avoid any possible misunderstanding about what is meant by having trouble *accepting* the death and emphasizes the emotionally distressing aspect of this experience.

*IGQ Item 5: Feeling sad or emotionally numb*.	
Are these feelings related to your loss?	The *ICD‐11* states that PGD “is differentiated from depressive episode because symptoms are specifically focused on the loss of the loved one,” and this allows this to be verified.

*Note*: ICD‐11 = International Statistical Classification of Diseases and Related Health Problems (11th rev.).

For the IGQ‐CC, each symptom item was presented individually to respondents on a screen, and if the respondent indicated they experienced a given symptom at any level (i.e., a score of 1 or higher), the clinical check was presented on the same screen below the symptom item. Table 1 shows each IGQ item and clinical check, which are answered on a “yes” or “no” basis, along with a rationale for the wording of each clinical check. It should be noted that participants could not amend their initial response to the symptom item after being presented with the clinical check. Generally, the clinical checks were designed to emphasize the core aspects of the symptom as described in the *ICD‐11*, provide clarity about the frequency and intensity of the symptom presentation, and/or ensure the relevance of the symptom to the bereavement experience.

The IGQ also assesses the degree to which symptoms exceed social, cultural, or religious norms (i.e., “Do you consider your grief to be worse [more intense and/or of longer duration] than what would be normally expected in your community or culture?”) and functional impairment associated with the symptoms (i.e., “Have these experiences caused problems in personal, family, social, educational, occupational, or other important areas of your life?”). To meet the diagnostic requirements for *ICD‐11* PGD: a person must report experiencing bereavement; the death related to this bereavement must have occurred 6 months ago or longer; at least one of the two core symptoms must be present; at least one of the three associated symptoms must be present; the participant must have responded answered “yes” or “I don't know” to the question on exceeding the expected cultural, social, or religious norms; and functional impairment must be present. To assess the effect of the clinical checks, a symptom was considered present based on a score of 2 (*moderately*) or higher on the Likert scale *and* an answer of “yes” on the follow‐up clinical check.

### Data analysis

All participants who did not report the loss of a loved one or reported that bereavement happened within the last 6 months were excluded from the analysis. The distribution of responses for the IGQ items is presented as counts and percentages along with endorsement rates. The counts and percentages of participants who endorsed each item were compared to those who endorsed each item *and* responded “yes” to the clinical check. The percentage decrease was calculated as:

$$\left(\mathrm{Endorsement}\%-\mathrm{CC}\%/\mathrm{Endorsement}\%\right)*100.$$

Next, the proportions of participants who met the core and associated symptom cluster requirements were compared with and without the application of the clinical checks, along with the overall *ICD‐11* PGD rates. All comparisons were made using the McNemar *Z* test, which is appropriate for comparing paired‐samples proportions.

## RESULTS

### Demographic and bereavement‐related characteristics

In total, 87.7% (*n* = 1,797) of participants reported a lifetime bereavement, and 84.0% (*n* = 1,723) were bereaved more than 6 months ago, thus satisfying the *ICD‐11* criteria for bereavement and timing. All analyses were based on these participants. The mean participant age for the bereaved sample was 43.30 years (*SD* = 13.04), and 52.1% (*n* = 897) were male. Most participants were married (*n* = 891, 51.7%), single (*n* = 347, 20.1%), or in a relationship (*n* = 246, 14.2%) with the remainder separated, widowed, or divorced (*n* = 239, 13.9%). Most participants had completed college/university (*n* = 1,003, 58.2%) or vocational school (*n* = 497, 28.8%) with others having completed general/secondary school (*n* = 183, 10.6%) or mandatory schooling (*n* = 40, 2.3%). Most participants were employed full‐ or part‐time (*n* = 1,148, 66.6%), 16.4% reported being unemployed (*n* = 282), and the remainder (*n* = 293, 17.0%) were students, retired, or not working due to disability.

When asked to identify the bereavement that caused the most distress, the most common response was the death of a parent (*n* = 803, 46.6%). The most common time frame for the death that caused this bereavement was “more than 10 years ago” (*n* = 684, 39.7%). The most common causes of death were “anticipated natural death” (*n* = 807, 46.8%) and “unexpected natural death” (*n* = 598, 34.7%). Additional bereavement‐related information is available in Supplementary Tables S1–S4.

### Item responses and endorsement rates

Table 2 presents item responses and endorsement rates both with and without the corresponding clinical checks. Symptom endorsements without clinical checks ranged from 34.2% (“feeling guilty or angry”) to 73.3% (“longing/yearning for the deceased”). Overall, endorsement rates were slightly higher for the two core symptoms than the three associated symptoms. The use of the clinical checks reduced endorsement rates for every symptom, with updated endorsement rates ranging from 23.6% (“feeling guilty or angry”) to 59.7% (“trouble accepting the loss”). The percentage decreases were noticeably higher for the two core PGD symptoms of yearning/longing and preoccupation (50.1% and 53.6%, respectively) compared to the three associated symptoms, which ranged from 10.8% to 31.0%.

**TABLE 2:** Frequency of item responses, endorsement, and clinical checks (CCs) for the International Grief Questionnaire

	**0 (*not at all*)**		**1 (*a little bit*)**		**2 (*moderately*)**		**3 (*quite a bit*)**		**4 (*extremely*)**		**Endorsement**		**Endorsement and CC**		**Decrease**
**Item**	*n*	%	*n*	%	*n*	%	*n*	%	*n*	%	*n*	%	*n*	%	%
1. Yearning	192	11.1	261	15.1	514	29.8	547	31.7	209	12.1	1,270	73.7	634	36.8	50.1
2. Thinking too much	286	16.6	398	23.1	554	32.2	355	20.6	130	7.5	1,039	60.3	483	28.0	53.6
3. Feeling guilty or angry	739	42.9	394	22.9	316	18.3	204	11.8	70	4.1	590	34.2	407	23.6	31.0
4. Trouble accepting	230	13.3	340	19.7	425	24.7	478	27.7	250	14.5	1,153	66.9	1,028	59.7	10.8
5. Feeling sad/numb	380	22.1	471	27.3	416	24.1	338	19.6	118	6.8	8,72	50.6	767	44.5	12.1

**TABLE 3:** Rates of probable prolonged grief disorder

**Variable**	***n***	**%**
Core symptom criteria	1,343	77.9
Core symptom criteria & clinical check	706	41.0
Associated symptom criteria	1,271	73.8
Associated symptom criteria & clinical check	1,163	67.5
Core symptom criteria and associated symptom criteria	1,122	65.1
Core symptom criteria, associated symptom criteria, and clinical check	626	36.3
Core symptom criteria, associated symptom criteria, and FI	273	15.8
Core symptom criteria, associated symptom criteria, FI, clinical check	201	11.7
Core symptom criteria, associated symptom criteria, FI, culture, and clinical check	234	13.6
Core symptom criteria, associated symptom criteria, FI, and culture; no clinical check	176	10.2

*Note*: FI = functional impairment criterion met; Culture = cultural criterion met.

Table 3 shows that at the symptom‐cluster level, significantly fewer people met the core (77.9% vs. 41.0%), McNemar's *Z* = 25.20, *p* < .001, and associated (73.8% vs. 67.5%), McNemar's *Z* = 10.30, *p* < .001, symptom requirements when the clinical checks were used. In line with the item‐level changes, the percentage reduction was larger for the core symptom cluster (47.4%) than the associated symptom cluster (8.5%).

At the disorder level, 13.6% (*n* = 234) of participants met the *ICD‐11* diagnostic requirements for PGD without the clinical checks, and this dropped to 10.2% (*n* = 176) with the clinical checks, McNemar's *Z* = 7.48, *p* < .001. This represents a 24.8% reduction in the number of people who met the diagnostic requirements for *ICD‐11* PGD when the clinical checks were applied.

## DISCUSSION

This proof of principle study was carried out to investigate if item‐level clinical checks that are a routine feature of clinical interviews can be embedded within a self‐report measure of *ICD‐11* PGD and to determine what effect they have on symptom endorsements and disorder prevalence. It is important to stress prior to discussing the findings that the clinical checks used in this study are not necessarily intended to be the final and immutable checks for the IGQ nor is the use of clinical checks relevant only in the case of PGD. Our intention is that these findings represent the beginning of a larger discussion in the scientific community about how assessments of psychopathology can improve. We invite all interested parties to consider what revisions can be made to the clinical checks used here for PGD symptoms assessed by the IGQ at this survey page: https://app.onlinesurveys.jisc.ac.uk/s/ulster/clinical‐checks

We selected *ICD‐11* PGD to test this approach for several reasons. First, PGD is a new and somewhat controversial disorder, and some researchers and clinicians have expressed concerns about the potential to pathologize normal psychological responses to a routine and difficult life event (Cacciatore & Frances, 2022). Perhaps more than any other disorder, differentiating “psychopathological” responses from “normal” reactions is of utmost importance. Second, and relatedly, because bereavement is often associated with some psychological distress, the potential for participants who are completing a self‐report questionnaire to erroneously report their “normal” distress as clinically relevant psychopathology is high. If, as many suspect, self‐report measures can capture higher‐than‐expected levels of symptomatic distress in some respondents and are, therefore, prone to producing false‐positive diagnoses (Bui et al., 2015), then this may be most likely to occur in the case of PGD. As such, PGD offers an ideal context in which to study the effects of clinical checks within a self‐report questionnaire. Third, as PGD is a new disorder in the *ICD‐11*, measures of this disorder, such as the IGQ, are in their infancy and are ideal candidates for testing and adapting.

Readers may well question if the clinical check we used in this study were the best possible; indeed, readers are invited to question the relevance of each and suggest alternatives, but our findings indicate that clinical checks can be implemented within a self‐report measure in a reasonably simple way and that they have a notable effect. Specifically, the endorsement rates for all five symptoms significantly declined with the addition of a single follow‐up statement answered on a “yes” or “no” basis. The implementation of these checks was easy in the current study, which collected data online. This gave us control and allowed us to fix a respondent's initial response to the symptom measure before presenting the clinical check. We did this to obtain a clear assessment of symptom endorsements with and without the use of clinical checks. When using pen‐and‐paper questionnaires, this will not be possible, but allowing a person to correct, update, or modify their symptom rating after completing the clinical check may be sensible. This would, in a way, mimic the process in a clinical interview where the interviewer can amend an initial rating based on newly obtained information. This is a subject for future research and consideration.

Our findings indicate that the clinical checks had the largest impact on the two core *ICD‐11* PGD symptoms, reducing endorsement rates by around 50% for each, and had less of an impact on the three associated symptoms, reducing endorsement rates by approximately 10%, 12%, and 30% for a given symptom. It's difficult to say why the clinical checks had a greater effect on the core symptoms than the associated symptoms. The proportion of participants who initially endorsed the two core symptoms was slightly higher than the proportion that endorsed three associated symptoms, so these symptoms may be more susceptible to being endorsed by individuals experiencing nonclinically relevant distress. The clinical checks for the core symptoms emphasized the need for these experiences to have occurred persistently and pervasively, and this may have led to the removal of many people who had these experiences only fleetingly or sporadically. It is also possible that the clinical checks for the associated symptoms were less well‐conceived than those for the core symptoms or that the checks for the core symptoms were too strict. These are speculative interpretations, and only replication will indicate if this is a consistent effect.

Despite the clinical checks having substantial effects at the item level, the effect at the diagnostic level was more modest. The proportion of participants who met the diagnostic requirements dropped by 3.4% in absolute terms (i.e., from 13.6% to 10.2%) when the clinical checks were used, equating to a relative reduction in cases of about 25%. There are different ways to interpret this result. On one hand, the clinical checks could be viewed as having an important and clinically significant effect, and a reasonable interpretation would be that the checks are eliminating what might otherwise be probable false‐positive cases. On the other hand, the change in the overall diagnostic rates was not so dramatic as to call into question the entire enterprise of estimating prevalence via self‐report. Arguably, the clinical checks are doing enough to warrant their inclusion in a self‐report measure when the goal is to estimate disorder prevalence and not so much that they undermine the validity of self‐report measurements without clinical checks. Obviously, what we do not know is how the prevalence estimates with and without the clinical checks compare to estimates obtained via a clinical interview.

Whether clinicians or researchers use clinical checks might depend on their goals. If one is interested in measuring symptom severity or maximizing diagnostic sensitivity (i.e., for initial screening purposes), standard self‐report measures would be ideal. However, if one is interested in estimating the prevalence rate of a given disorder in a population or balancing concerns about sensitivity and specificity when engaging in clinical screening processes, adding clinical checks to a self‐report measure could be advantageous. The addition of clinical checks would allow for severity scores to be calculated in the normal way and prevalence estimates to be obtained with more confidence than when using self‐report data in the normal way. Potentially, the self‐report measure with clinical checks fills the rather large gap between the conventional thinking regarding self‐report for surveys and interviews for diagnosis.

These results should, of course, be interpreted cautiously given some study limitations. First, there were no clinical interview data with which to compare the self‐report scores with and without the clinical checks. Therefore, we cannot speak to how the checks directly impacted prevalence estimates. Although it is reasonable to suspect that this is indicative of a decrease in false positives, it is also possible that prevalence decreased due to true positive cases questioning their initial responses. Second, the data were collected from a general population sample of adults living in Ukraine during a time of war. Studying bereavement and grief in such a unique context is timely and important, but the generalizability of the findings is unclear. Third, the study authors formulated the clinical checks. As this was conceived as a proof of principle study, we did not seek input from an extensive pool of researchers and clinicians with expertise in bereavement and grief nor did we pilot the clinical checks with bereaved individuals and seek feedback. Fourth, a case was made for the clinical checks being quick and easy to complete, but there was no timing or feedback available in this study. There are two main future directions of research in this area. First, it is important that the performance of self‐report measures with clinical checks is compared to clinical interviews. Second, if this approach is found to repeatedly produce valid and reliable scores, then it could be used for the assessment of other stress‐related disorders, such as PTSD and CPTSD, as well as other psychological disorders that are routinely assessed in large‐scale epidemiological studies.

What we have suggested here is not so much an alternative to the standard assessment methods of clinical interviews and self‐report instruments but rather an addition to these methods that might incorporate some of the best features of both and, thus, offset some of the worst features of both. By modifying standard self‐report measures with the types of checks that are commonplace in clinical interviews, it may be that clinicians and researchers will have access to a method that will allow them to assess disorder prevalence in a way that is inexpensive, quick, easy to implement, and easy to complete.

## OPEN PRACTICES STATEMENT

The study reported in this article was not formally preregistered. The data that support the findings of this study are available on request from the corresponding author at M.Shevlin@ulster.ac.uk. The data are not publicly available due to privacy or ethical restrictions.

## Supporting information

SUPPORTING INFORMATION
