# Supplementary material for: Assessing self‐reported prolonged grief disorder with “clinical checks”: A proof of principle study
Source: J Trauma Stress. 2024 Aug 19;38(1):174–80. doi: 10.1002/jts.23100 (PMC11791873; doi:10.1002/jts.23100)
Supplement: Supplementary file 1 — SUPPORTING INFORMATION [file JTS-38-174-s001.docx]

Supplementary Table S1. Time Since Bereavement.

|  | Frequency | Percent | Cumulative Percent |
| --- | --- | --- | --- |
| 6 months to a year ago | 88 | 5.1 | 5.1 |
| 1-2 years ago | 181 | 10.5 | 15.6 |
| 2-3 years ago | 146 | 8.5 | 24.1 |
| 3-5 years ago | 244 | 14.2 | 38.2 |
| 6-10 years ago | 380 | 22.1 | 60.3 |
| More than 10 years ago | 684 | 39.7 | 100.0 |
| Total | 1723 | 100.0 |  |

Supplementary Table S2. Frequency of Social Contact with the Bereaved in the Previous Year (in person, telephone calls, video calls, text messages, etc).

|  | | Frequency | Percent | Cumulative Percent |
| --- | --- | --- | --- | --- |
|  | Every day | 613 | 35.6 | 35.6 |
|  | Almost every day | 348 | 20.2 | 55.8 |
|  | Several times a week | 290 | 16.8 | 72.6 |
|  | Several times a month | 254 | 14.7 | 87.3 |
|  | A few times in the year | 173 | 10.0 | 97.4 |
|  | Not at all during that year | 45 | 2.6 | 100.0 |
|  | Total | 1723 | 100.0 |  |

Supplementary Table S3. Nature of the death?

|  | | Frequency | Percent | Cumulative Percent |
| --- | --- | --- | --- | --- |
|  | Anticipated natural death (e g , death after a period of terminal illness) | 807 | 46.8 | 46.8 |
|  | Unexpected natural death (e g , cardiac arrest, epileptic seizure, stroke, stillbirth) | 598 | 34.7 | 81.5 |
|  | Sudden unnatural death (e g , road traffic accident, murder or manslaughter, work accident) | 148 | 8.6 | 90.1 |
|  | Suicide | 30 | 1.7 | 91.9 |
|  | Died in the war (Death directly as a result of military operations (during combat clashes as part of military formations | 55 | 3.2 | 95.1 |
|  | Other | 85 | 4.9 | 100.0 |
|  | Total | 1723 | 100.0 |  |

Supplementary Table S4. Death that affected participant the most?

|  | | Frequency | Percent | Cumulative Percent |
| --- | --- | --- | --- | --- |
|  | Child | 64 | 3.7 | 3.7 |
|  | Partner or spouse | 68 | 3.9 | 7.7 |
|  | Parent | 803 | 46.6 | 54.3 |
|  | Brother or sister | 73 | 4.2 | 58.5 |
|  | Grandparent | 484 | 28.1 | 86.6 |
|  | Uncle or aunt | 55 | 3.2 | 89.8 |
|  | Cousin | 36 | 2.1 | 91.9 |
|  | Niece or nephew | 8 | .5 | 92.3 |
|  | Close friend | 100 | 5.8 | 98.1 |
|  | Colleague | 13 | .8 | 98.9 |
|  | Acquaintance | 19 | 1.1 | 100.0 |
|  | Total | 1723 | 100.0 |  |
